# Blocking autophagy enhances the apoptotic effect of 18*β*-glycyrrhetinic acid on human sarcoma cells via endoplasmic reticulum stress and JNK activation

**DOI:** 10.1038/cddis.2017.441

**Published:** 2017-09-21

**Authors:** Shuying Shen, Menglu Zhou, Kangmao Huang, Yizheng Wu, Yan Ma, Jiying Wang, Jianjun Ma, Shunwu Fan

**Affiliations:** 1 Department of Orthopaedic Surgery, Sir Run Run Shaw Hospital, Medical College of Zhejiang University, Sir Run Run Shaw Institute of Clinical Medicine of Zhejiang University, 3 East Qingchun Road, Hangzhou, Zhejiang Province, China; 2 Institute of Biochemistry, College of Life Science, Zhejiang University, Hangzhou, Zhejiang Province, China

## Abstract

Sarcoma, a rare form of cancer, is unlike the much more common carcinomas as it occurs in a distinct type of tissue. The potent antitumor effects of 18*β*-glycyrrhetinic acid (GA), a novel naturally derived agent, have been demonstrated in various cancers. However, the effect of GA on human sarcoma, and the underlying mechanisms, remain to be elucidated. In the current study, we show that GA inhibits sarcoma cell proliferation by inducing G0/G1-phase arrest. Exposure to GA resulted in the activation of caspase-3, -8, and -9, indicating that GA induced apoptosis through both extrinsic and intrinsic pathways. In addition, the autophagy pathway, characterized by the conversion of LC3-I to LC3- II, was activated, resulting in increased Beclin-1 protein levels, decreased p62 expression, and stimulation of autophagic flux. The present findings showed that GA stimulated autophagy by inducing endoplasmic reticulum (ER) stress via the IRE1–JNK pathway. Our data supported the prosurvival role of GA-induced autophagy when the autophagy pathway was blocked with specific chemical inhibitors. Finally, GA markedly reduced sarcoma growth, with little organ-related toxicity, *in vivo*. The present results suggest that the combination of GA with a specific autophagy inhibitor represents a promising therapeutic approach for the treatment of sarcoma.

There are two general categories of sarcoma: soft-tissue sarcoma, such as fibrosarcoma, and primary bone sarcoma, of which the most common type is osteosarcoma. Both categories are rare, with poor outcome.^1^ Doxorubicin is considered the first-line chemotherapeutic drug for soft-tissue sarcomas. However, therapeutic doses of doxorubicin have been shown to result in cardiotoxicity.^2^ Osteosarcoma, a highly malignant form of cancer, originates in the osteogenic mesenchymal matrix whose cells are capable of forming osseous, osteoid, cartilaginous, and fibrous tissues.^3^ This form of cancer is well known for its aggression and tendency to form early distant metastases; among these, pulmonary metastasis foci, being the most common, are observed in over 80% of cases.^4, 5^ Current therapeutic strategies against the disease, such as chemotherapy and large-scale surgery, do not significantly increase postsurgical 5-year survival rates.^6^ Therefore, the development of novel treatment approaches and therapeutic drugs for sarcoma is essential.

The 18*β*-glycyrrhetinic acid (GA), the major bioactive component in licorice root, exerts antioxidative, anti-inflammatory, and anticancer effects;^7, 8, 9^ in patients with ovarian and breast cancers, the GA-containing Hongdoushan capsule (CHC) has been clinically used as an effective therapeutic strategy.^10^ Furthermore, previous studies have demonstrated that GA suppresses tumor cell growth in breast,^8^ lung,^11^ and gastric^12^ cancers. In addition, GA has been shown to significantly reduce cisplatin-related nephrotoxicity, both in terms of the occurrence rate and severity, via the Nrf2/NF-*κ*B signaling pathway in BALB/c mice.^13^ However, further studies are required to elucidate the mechanisms by which GA inhibits tumorigenesis *in vivo* and *in vitro*.

Cell death may generally be classified as necrosis, autophagy, apoptosis, cornification, and the so-called atypical cell death modalities.^14^ Apoptosis, a type-I programmed cell death (PCD), plays a role in the effect of chemotherapies against various carcinomas.^15^Autophagy, a type-II PCD, involves the caspase-independent cell death pathway.^16^ In some cellular settings, autophagy may serve as a cell death promoter, working in concert with apoptosis or as a back-up mechanism when the former is defective. However, in others, autophagy serves as a cell survival pathway via the suppression of apoptosis.^17^ It is not known whether GA induces apoptosis or autophagy; furthermore, the specific putative roles of these two forms of cell death and their interplay in GA-induced cell death in cancer remain to be determined.

Autophagy is regulated via the Akt/mammalian target of rapamycin (mTOR) pathway; accordingly, suppression of the Akt/mTOR cascade significantly increases autophagy. Rapamycin, a well-known mTOR inhibitor, is widely used as an inducer of autophagy.^18, 19, 20, 21^ The mitogen-activated protein kinase family also act as crucial autophagy mediators. Previous studies have demonstrated that autophagy may be induced by activation of extracellular signal-regulated kinase (ERK) through the action of numerous compounds.^22, 23^ Recent electron tomography analyses have demonstrated that autophagosome membranes are connected to the ER, thereby suggesting a direct connection between the ER and autophagy.^24, 25^ C-Jun N-terminal kinase (JNK) further exerts important effects on endoplasmic reticulum (ER) stress-induced autophagy; in JNK pathway-deficient *in vitro* and *in vivo* models, ER stress-induced cell death is significantly elevated in the absence of autophagy.^26, 27^

In the present study, we demonstrated the inhibitory effect of GA on sarcoma cell lines *in vitro* and *in vivo*, and explored the underlying molecular mechanisms by which GA elicits these effects. We additionally confirmed that GA induces cytoprotective autophagy in sarcoma cells via IRE1*α*-JNK/c-jun cascade activation, and that inhibition of autophagy or the JNK pathway increases GA-induced inhibitory effects and apoptosis on sarcoma cells.

## Results

### GA inhibits the proliferation of sarcoma and induces G0/G1-phase arrest by regulating cell cycle-regulated proteins

**Figure 1:**
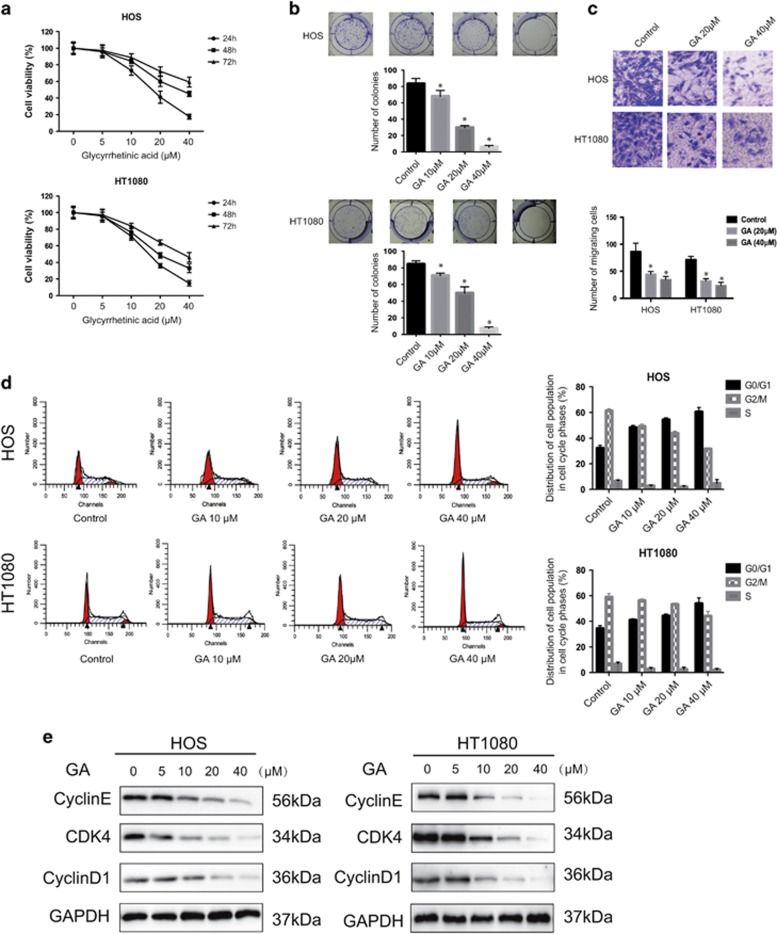
Cytotoxic effects and G0/G1-phase arrest resulting from GA treatment in sarcoma cells. (**a**) The antiproliferative effect of GA on sarcoma cell lines was determined by MTT. Cells were treated with various concentrations of GA for 24, 48, and 72 h. Control group contained 0.1% DMSO. Data represented the mean of five replicates. Each experiment was performed in triplicate. (**b**) Colony formation assay of HOS and HT1080 cells with control or GA. Cells were treated with GA at varying concentrations for ∼14 days when the cells grew to visible colonies. (**c**) GA inhibited sarcoma cell invasion in transwell assay. The bottom chambers of the transwells were filled with 600 *μ*l DMEM containing various growth factors, whereas the top chambers were seeded with 4 × 10^4^ sarcoma cells in DMEM and treated with different concentrations of GA for 24 h. Cells that migrated through the membrane were stained. Number of migrating cells was counted. *P<0.05, significantly different compared with control. (**d**) GA induced G0/G1-phase arrest. Cells were treated with control or GA for 24 h and analyzed by flow cytometry. The percentage of cell cycle distribution was presented as the mean±S.D. from three independent experiments. (**e**) HOS and HT1080 cells were treated with GA for 24 h. The expressions of cell cycle-regulated proteins were measured by western blot. *P<0.05, significantly different compared with control

In order to investigate the effect of GA on cell growth, HOS and HT1080 cells were exposed to GA at various concentrations for 24, 48, and 72 h (Figure 1a). The IC_50_ values were 45.845 *μ*M (24 h), 34.81 *μ*M (48 h), and 24.436 *μ*M (72 h) for HOS cells; IC_50_ values were 34.013 *μ*M (24 h), 25.656 *μ*M (48 h), and 20.554 *μ*M (72 h) for HT1080 cells. Colony formation assays showed that fewer colonies were formed following GA treatment (Figure 1b). These results demonstrated that GA inhibits proliferation of sarcoma in a dose- and time-dependent manner. Cell migration is essential for sarcoma cells in metastasis. Therefore, we performed transwell assays to evaluate the ability of sarcoma cells to pass through the membrane barriers of the transwell in the presence of various concentrations of GA. As shown in Figure 1c, GA significantly inhibited the migratory activities of sarcoma cells in a concentration-dependent manner.

In order to determine whether GA inhibits cell proliferation via induction of cell cycle arrest, we examined cell cycle stages in cells treated with GA. As shown in Figure 1d, GA treatment in HOS and HT1080 cells resulted in an accumulation of cells in G0/G1 phase, and a corresponding decrease in cells in G2/M and S phases. To elucidate the mechanisms underlying this phenomenon, we measured protein expression cell cycle regulators. Results indicated that GA downregulates the expression of Cyclin E, CDK4, and Cyclin D1 (Figure 1e), but has no effect on their mRNA levels (Supplementary Figure S1). These results clearly indicated that GA induces G0/G1-phase arrest by altering the expression of G0/G1 cell cycle regulators.

### GA induces apoptosis of sarcoma cells

**Figure 2:**
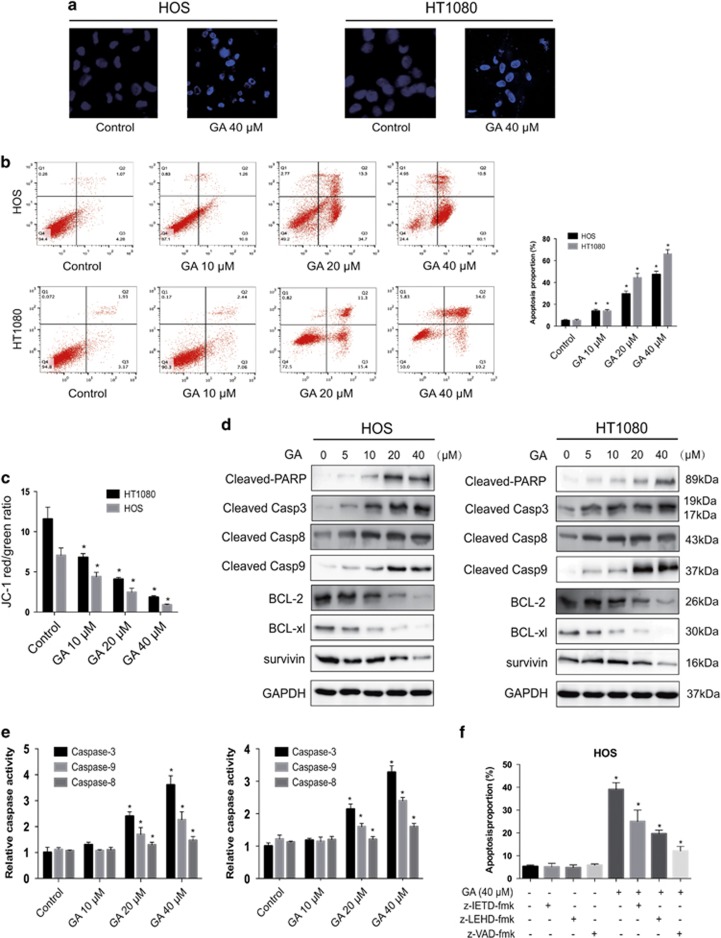
Evidence that GA induces apoptosis in sarcoma cells. (**a**) Apoptotic morphological changes were evaluated by fluorescent microscopy using Hoechst 33342 staining. Arrows indicate chromatin condensation and DNA fragmentation. Bar, 50 *μ*m. (**b**) HOS and HT1080 cells treated with GA were stained with annexin V-FITC/PI and analyzed by flow cytometry. The chart illustrates apoptosis proportion from three separate experiments. (**c**) The mitochondrial membrane potential was measured with JC-1 fluorescent probe and assessed by flow cytometry. The chart illustrates changes of JC-1 red/green rate from three independent experiments. (**d** and **e**) Cells were treated with various concentrations of GA for 24 h. The expressions of cleaved PARP, caspase-3, -8, -9, BCL-2, BCL-XL, and survivin were determined by western blot. (**e**) Caspase activity assay of cells treated with various concentrations of GA for 24 h. (**f**) HOS cells were incubated with or without GA for 24 h after 2 h of pretreatment with caspase inhibitors, z-IETD-fmk (10 *μ*M), z-LEHD-fmk (40 *μ*M), or z-VAD-fmk (20 *μ*M). Then, cells were stained with annexin V-FITC/PI and analyzed by flow cytometry. Results are expressed as the mean±S.D. from three independent experiments. *P<0.05 *versus* control, ^#^P<0.05 *versus* GA treatment

In order to determine whether apoptosis is responsible for the inhibition of cell growth in response to GA, we further explored the effects of GA on apoptosis and cell death in HOS cells. Hoechst 33342 staining was used to estimate GA-dependent changes in cell morphology. Results indicated that GA treatment for 24 h induced cell shrinkage, chromatin condensation, and nuclear fragmentation (Figure 2a). Flow cytometric analysis using annexin V- FITC/PI staining was performed. The apoptosis rate of untreated cells was negligible; however, 24 h following GA exposure in sarcoma cells, we observed a dose-dependent increase in both early and late apoptotic cells (Figure 2b). Next, in order to confirm the involvement of mitochondria GA-associated cell apoptosis, the fluorescent mitochondrial probe JC-1 was used to measure the mitochondrial membrane potential (MMP). As shown in Figure 2c and Supplementary Figure S1, MMP was sharply reduced following GA treatment, suggesting that mitochondrial depolarization was induced by exposure to GA in sarcoma cells.

### GA induces caspase-dependent apoptosis through the intrinsic pathways

In mammalian cells, apoptosis may be induced by extra- or intracellular stimuli, triggering the extrinsic (via cell-surface death receptors) or intrinsic pathways (via mitochondrial signaling), respectively.^28^ In order to determine whether GA-induced apoptosis is mediated by extrinsic or intrinsic pathways, we investigated the expression of downstream apoptotic proteins by western blot. Caspase-8 and -9 act as initiator caspases in the extrinsic and intrinsic (mitochondrial) apoptosis pathways, respectively. As shown in Figure 2d, a significance increase in activation of cleavage caspase-3, -8, and -9, as well as of PARP, was observed. However, the expression of Bcl-2, Bcl-xl, and survivin was reduced. Taken together, these data indicated that GA induces cell apoptosis via activation of both the extrinsic and intrinsic pathways. In order to confirm these results, we performed caspase activity assays. Activities of caspase-3, -8, and -9 increased with escalating doses of GA (Figure 2e). We further investigated the roles of these caspases using z-VAD-fmk, z-IETD-fmk, and z-LEHD-fmk. As expected, we observed a moderate inhibitory effect for z-IETD-fmk and z-LEHD-fmk in GA-induced apoptosis; z-VAD-fmk demonstrated a more potent inhibitory effect (Figure 2f). These data confirmed that GA stimulates caspase-dependent apoptosis via activation both extrinsic and intrinsic pathways.

### GA triggers autophagy in sarcoma cells

**Figure 3:**
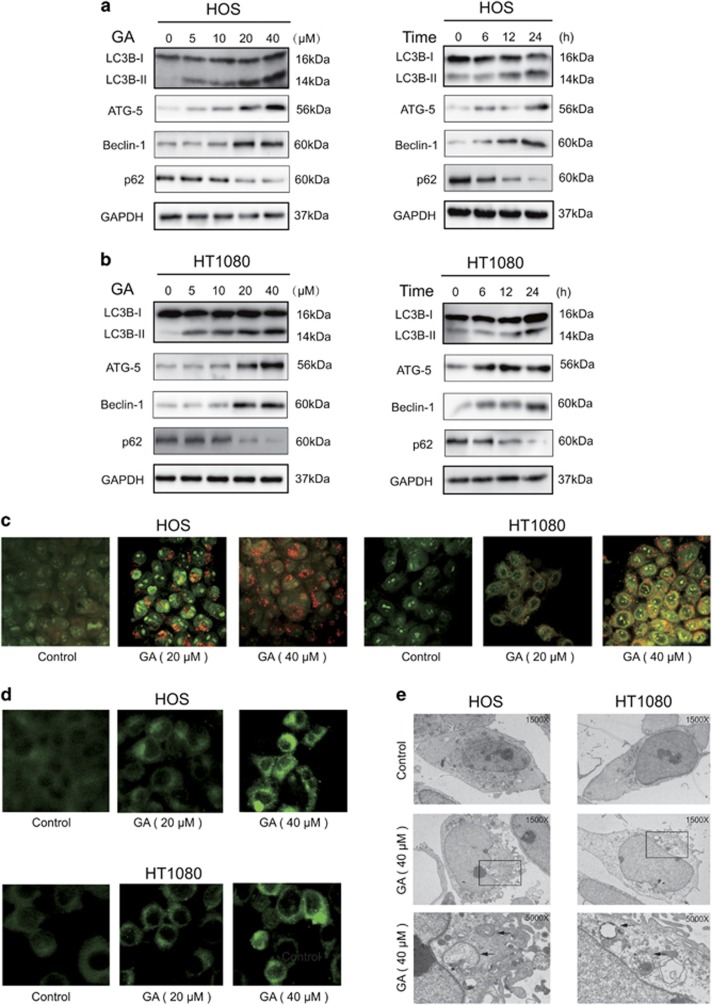
GA induces autophagy. (**a** and **b**) Cells were treated with various concentrations of GA for 24 h or incubated with GA (40 *μ*M) for different hours. The level of LC3B, Atg5, Beclin-1, and p62 was measured by western blot. (**c**) Cells treated with or without GA for 24 h were collected and stained with acridine orange. Representative images of acridine orange-stained cells captured by fluorescent microscopy (× 400) are shown. Bar, 50 *μ*m. (**d**) Sarcoma cells were treated with GA for 24 h and stained by MDC. (**e**) Transmission electron microscopy was utilized to observe the formation of autophagosome and ultrastructural change of nucleus. Arrows indicate autophagosomes containing intact and degraded cellular debris. Asterisks indicate nuclear condensation. Bar, 1 *μ*m

The occurrence of autophagy in GA-treated HOS and HT1080 cells was investigated by measurement of LC3-I to LC3-II conversion, a hallmark of autophagy. In addition, protein levels of Beclin-1, a key regulator of autophagy formation,^29^ as well as p62, a selective target of autophagy, was measured. As shown in Figures 3a and b, protein levels of Beclin-1 were elevated following GA treatment; furthermore, conversion of LC3-I to LC3-II was significantly enhanced. The expression of the autophagy-related protein 5 (Atg5) also exhibited continuous increase following treatment with GA. Previous studies have shown that p62 is degraded during the autophagy process;^14^ here, p62 levels were significantly reduced after 24 h of treatment with GA. Furthermore, GA treatment led to the accumulation of bright red acidic vesicles resembling autolysosomes (Figure 3c). To confirm occurrence of autophagy, we measured the incorporation of MDC, a marker for mature autophagic vacuoles (AVs) such as autophagolysosomes, in sarcoma cells. GA treatment significantly increased the level of MDC-stained AVs in both sarcoma cell types used (Figure 3d). Transmission electron microscopy (TEM) was used to directly observe autophagosome formation. As shown in Figure 3e, we observed numerous large autophagic vacuoles in the cytoplasm with degraded vacuolar contents, concurrent with apoptotic chromatin condensation. These results provided evidence for a role of GA in regulation of autophagosome formation in sarcoma cells.

### GA induces autophagy via JNK/c-jun pathway activation in sarcoma cells

**Figure 4:**
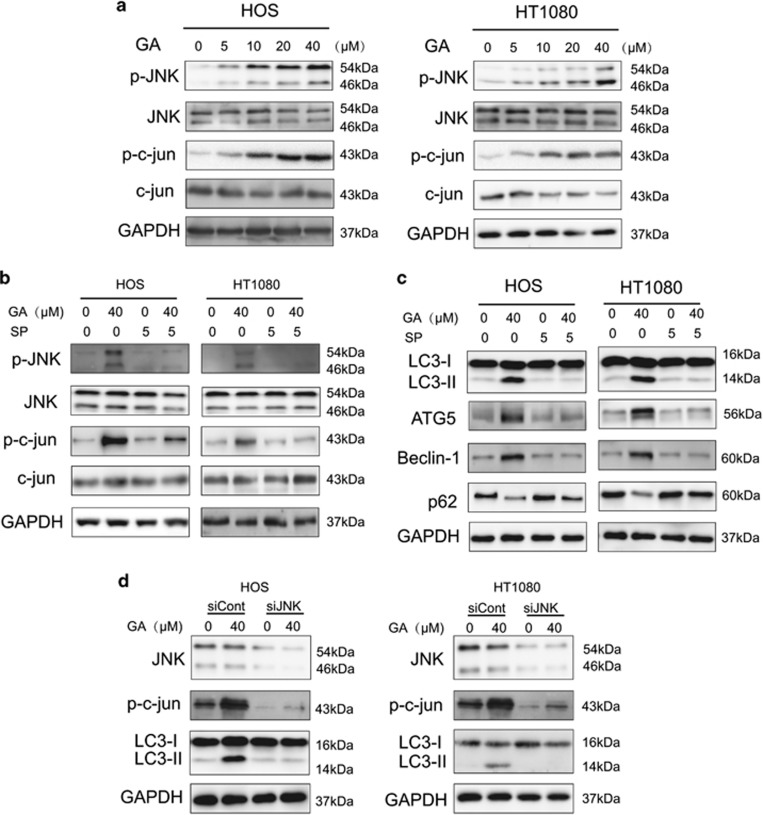
GA activates autophagy through JNK/c-jun signal pathway. (**a**) Cells were treated with various concentrations of GA for 24 h. The expression of p-JNK, JNK, p-c-Jun, and Jun were analyzed by western blotting. (**b**) Sarcoma cells was preincubated with SP600125 (30 *μ*M) for 2 h and then treated with GA for 24 h. Levels of p-JNK, JNK, p-c-jun, and c-jun were analyzed by western blotting. (**c**) Sarcoma cells was preincubated with SP600125 (30 *μ*M) for 2 h and then treated with GA for 24 h. Levels of Atg5, Beclin-1, and p62 were analyzed by western blotting. (**d**) After knockdown of JNK by siRNA, HOS and HT1080 cells were incubated in GA (40 *μ*M) for 24 h. Levels of JNK, p-c-Jun, and LC3-I/II were analyzed by western blotting

The JNK/c-jun pathway plays a pivotal role in autophagy, and activation of JNK/c-jun induces autophagy under diverse conditions.^30, 31^ Therefore, we investigated whether GA was capable of inducing autophagy via the JNK/c-jun cascade. As shown in Figure 4a, GA induced phosphorylation of JNK and c-Jun in a concentration-dependent manner in both HOS and HT1080 cells.

The JNK/c-jun pathway inhibitor SP600125 was used to determine whether GA induces autophagy via JNK/c-jun activation.^32^ As shown in Figure 4b, GA-activated JNK and c-jun levels were decreased following pretreatment with SP600125 (10 *μ*M, 1 h). In addition, induction of autophagy markers such as LC3-II, ATG5, and Beclin-1 was also reversed following SP600125 treatment. This further suggested that GA induces autophagy via the JNK/c-jun pathway (Figure 4c). In order to confirm the role of JNK in GA-induced autophagy, JNK silencing was performed using siRNA. We observed a decrease in GA-induced LC3-II expression in both HOS and HT1080 cell lines (Figure 4d) following JNK inhibition. Therefore, activation of the JNK/c-jun cascade is pivotal for GA-induced autophagy in sarcoma cells.

### ER stress-stimulated JNK/c-jun activation is critical for GA-induced autophagy in sarcoma cells

**Figure 5:**
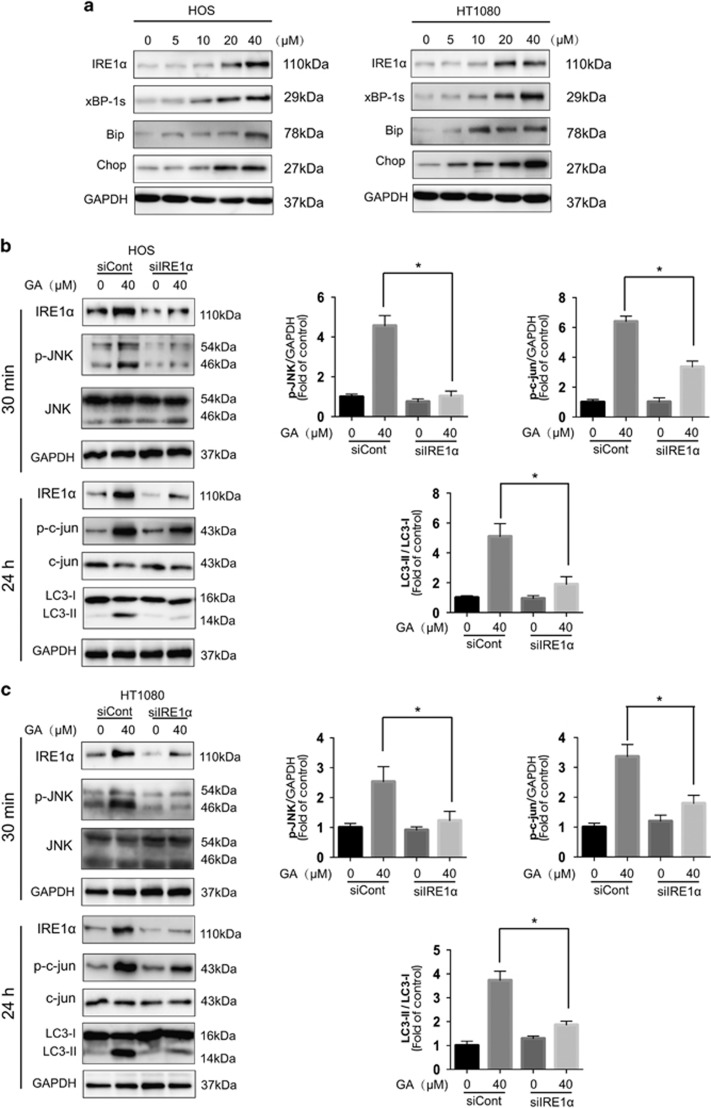
The IRE1*α*-JNK/c-jun pathway is essential for GA-induced autophagy in sarcoma cells. (**a**) HOS and HT1080 cells were treated with varying concentrations of GA for 24 h, and the ER stress-related protein expression was evaluated by western blot analysis. (**b** and **c**) HOS and HT1080 cells were infected with scramble or IRE1*α* siRNA and then incubated for 24 h. GA (40 *μ*M) was added to the cells for 30 min or 24 h. Protein expression related to the JNK/c-jun pathway and autophagy was detected by western blot analysis. *P<0.05

**Figure 6:**
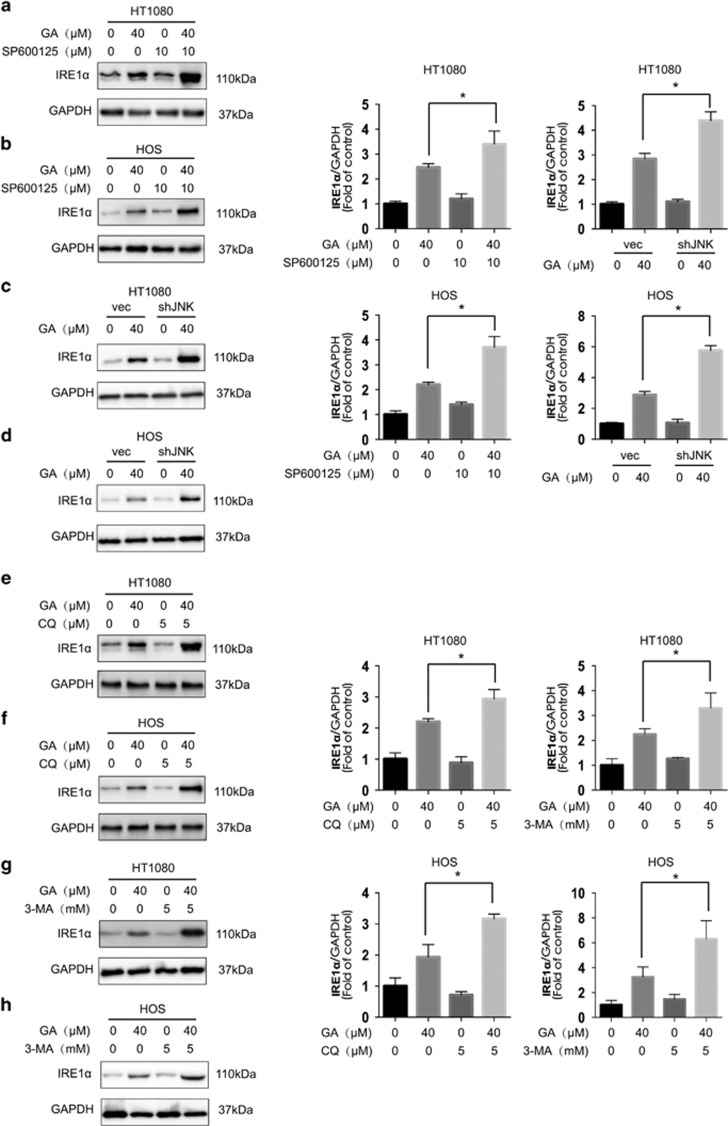
Inhibition of JNK/c-jun and autophagy increases GA-induced ER stress in sarcoma cells. (**a** and **b**) After suppression of the JNK/c-jun cascade by SP600125 (10 *μ*M, 1 h) pretreatment, HT1080 and HOS cells were treated with GA (40 *μ*M) for 24 h. Protein expression levels were detected by western blot analysis. *P<0.05. (**c** and **d**) After suppression of the JNK by shRNA transfection, HT1080 and HOS cells were treated with GA (40 *μ*M) for 24 h. Protein expression levels were detected by western blot analysis. *P<0.05. (**e** and **f**) HT1080 and HOS cells were treated with GA (40 *μ*M) for 24 h with or without pretreatment of CQ (5 *μ*M, 1 h). Western blot analysis was used to examine protein expression. *P<0.05. (**g** and **h**) HT1080 and HOS cells were treated with GA (40 *μ*M) for 24 h with or without pretreatment of 3-MA (5 mM, 2 h). Western blot analysis was used to examine protein expression. *P<0.05

Two classic ER stress inducers, thapsigargin and tunicamycin, have been found to stimulate autophagy.^27, 33^ In the current study, we found that GA-induced activation of JNK/c-jun activation was mediated by ER stress. Expression of the ER stress sensor IRE1*α*, as well as its downstream molecule XBP-1s, and the ER stress-related proteins Chop and Bip were increased after GA treatment (Figure 5a). IRE1*α* knockdown by siRNA decreased GA-induced autophagy, as evidenced by decreased LC3-II expression (Figures 5b and c). In addition, ER stress and JNK/c-jun was found to be correlated in HOS and HT1080 cells. Knockdown of IRE1*α* partly inhibited phosphorylation of JNK and c-jun after 30 min and 24 h of GA treatment, respectively (Figures 5b and c, Supplementary Figure S3). However, JNK knockdown, as well as inhibition of the JNK/c-jun pathway by SP600125, increased GA-induced ER stress in HOS and HT1080 cells (Figures 6a–d, Supplementary Figure S4) that may be attributed to inhibition of GA-induced autophagy. Combined treatment with GA and CQ or 3-MA also increased GA-induced ER stress (Figures 6e–h), suggesting that inhibition of autophagy results in increased levels of misfolded and damaged proteins in the cell that initiates the ER stress response.^27^ These results indicated that GA induces autophagy in HOS and HT1080 cells via the IRE1*α*-JNK/c-jun pathway, and that inhibition of JNK/c-jun or autophagy increases GA-induced ER stress.

### Protective autophagy during GA treatment

**Figure 7:**
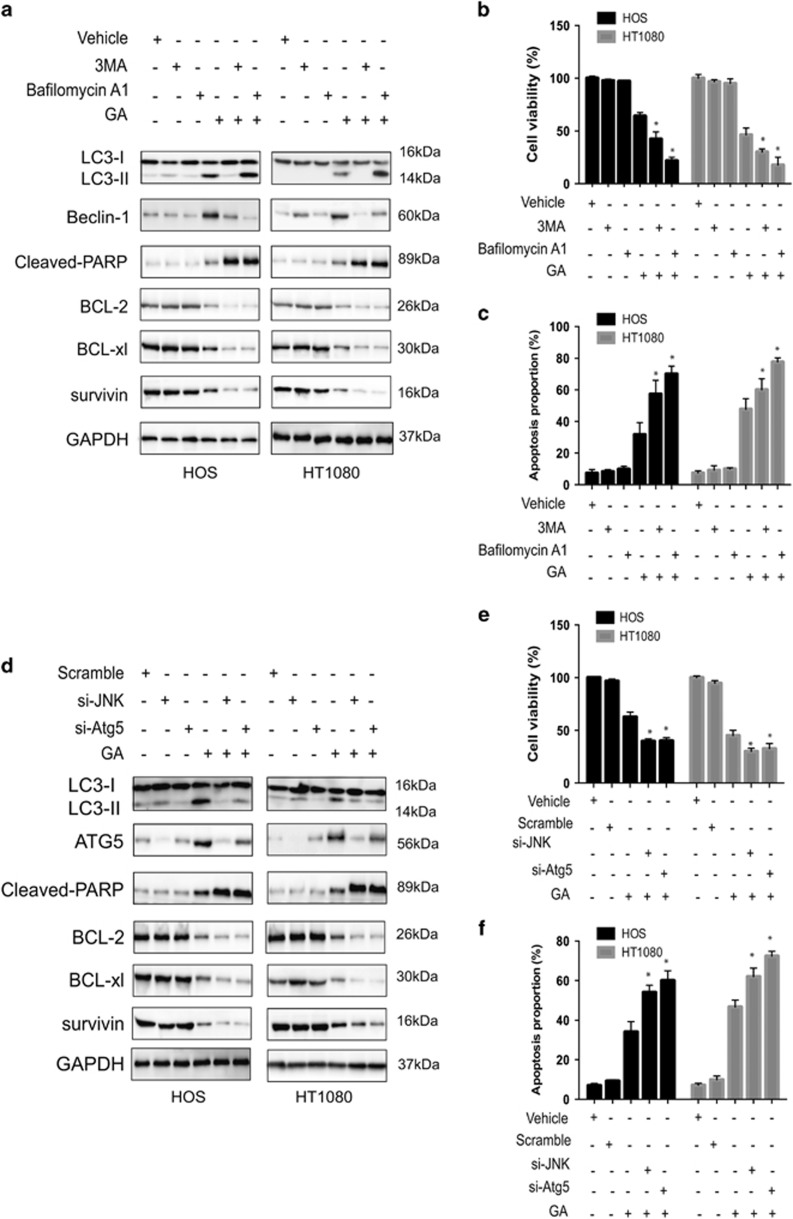
Inhibition of autophagy or JNK enhances GA-induced cell proliferative inhibition and apoptosis in sarcoma cells. (**a**) 3-MA or bafilomycin A1 was pretreated to HOS and HT1080 cells for 6 h before incubating in GA (40 *μ*M) for 24 h. Western blot analysis examined the protein expression level of Beclin-1, BCL-2, BCL-xl, survivin, cleaved-PARP, and LC3 conversion. (**b**) After pretreatment with 3-MA or bafilomycin A1 for 6 h, HOS and HT1080 cells were incubated in GA (40 *μ*M) for 24 h. Then, MTT assay was used to test cell viability to calculate the growth index. (**c**) Cells in (**b**) were analyzed by flow cytometry. Histograms were shown for analyzed cells (*n*=3). *P<0.05. (**d**) After knockdown of JNK or Atg5 by siRNA, HOS and HT1080 cells were incubated with 40 *μ*M of GA for 24 h. Western blot analysis examined the protein expression level of Atg5, BCL-2, BCL-xl, survivin, cleaved-PARP, and LC3 conversion. (**e**) After knockdown of JNK or Atg5 by siRNA, HOS and HT1080 cells were incubated in GA (40 *μ*M) for 24 h. Then, MTT assay was used to test cell viability to calculate the growth index. (**f**) Cells in (**e**) were analyzed by flow cytometry. Histograms were shown for analyzed cells (*n*=3). *P<0.05

It is known that autophagy can serve either as an alternative form of programmed cell death or a form of protection during critical periods.^34^ To determine whether autophagy induced by ER stress promotes cell survival or cell death in sarcoma cells, we used specific inhibitors of autophagy. Cells were treated with bafilomycin A1 that inhibits fusion of the autophagosome with the lysosome; we also examined the effect of 3-methyladenine (3-MA) that suppresses the activity of the class III PI3Ks. Sarcoma cells were pretreated with 10 mM 3-MA or 100 nM bafilomycin A1 for 6 h; neither agents exerted significant toxic effects on cells. The inhibitory effect of these drugs on autophagy was assessed using MTT and annexin V/PI staining. As compared with untreated cells, bafilomycin A1- or 3-MA-treated cells underwent extensive cell death after GA treatment (Figures 7b and c). In addition, Atg5 siRNA-transfected cells also exhibited significantly increased cell death and apoptosis rates in response to GA (Figures 7e and f). As the IRE1*α*-JNK/c-jun pathway plays an important role in GA-induced autophagy, we evaluated the effect of GA on cell growth and apoptosis via suppression of the JNK/c-jun pathway. As expected, GA inhibited cell growth inhibition, and an increase in apoptosis was found following JNK blockage in both HOS and HT1080 sarcoma cell lines (Figures 7d–f). Furthermore, both autophagy inhibition and JNK knockdown enhanced the level of apoptosis-related proteins following GA treatment (Figures 7a and d). These data suggested that ER stress-induced autophagy mediated by GA treatment exerts cytoprotective effects.

### GA inhibits growth of osteosarcoma *in vivo*

**Figure 8:**
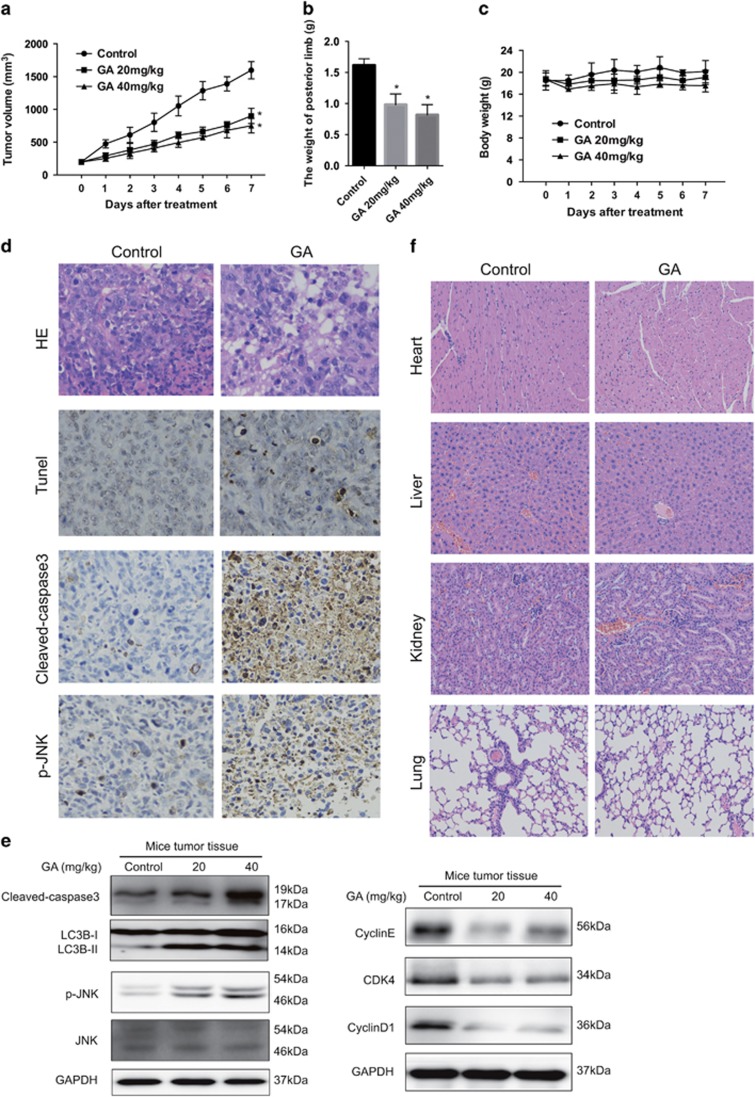
GA inhibits OS xenograft growth *in vivo*. HOS cells were orthotopically inoculated into the left tibia of BALB/c-nu mice. At 1 week after tumor inoculation, mice were randomly divided into two groups for treatment. Intraperitoneal administration of vehicle or GA (20 or 40 mg/kg) every other day for seven times. (**a**) Tumor volume was measured every week. (**b**) GA treatment resulted in significantly lower than control group. (**c**) Body weights were measured every week. (**d**) The apoptotic status of tumor tissues was assessed by TUNEL assay. H&E staining was used to evaluate the histology. The expression levels of cleaved caspase-3 and phospho-JNK were also examined by immunohistochemistry. Representative images were presented. Bar, 50 *μ*m. (**e**) The levels of cleaved caspase-3, LC3-I/II, phospho-JNK, total JNK, Cyclin E, CDK4, and Cyclin D1 in tumor xenograft tissues were measured by western blot. (**f**) No major organ-related toxicities was observed. H&E staining was used to evaluate the histology

In order to examine the antitumor effect of GA *in vivo*, an orthotopic OS model was established by intratibial injection of HOS cells into mice. Mice in the experimental group were injected with GA (2 mg/kg), whereas those in the control group received 5% dimethyl sulfoxide (DMSO) intraperitoneally every other day (seven times in total). As shown in Figures 8a and b, GA inhibited tumor growth. However, no significant decrease in body weight was observed in experimental mice (Figure 8c). Moreover, GA-treated tumor tissues showed significant increase in TUNEL-positive cells, and exhibited increased levels of cleaved caspase-3 and JNK phosphorylation (Figure 8d). As shown in Figure 8e, GA treatment led to elevated levels of cleaved caspase-3, LC3B-II, phospho-JNK, Cyclin E, CDK4, and Cyclin D1. In order to investigate the potential cytotoxic effects of GA on normal tissues, nontumor-bearing mice were intraperitoneally treated with GA; hematoxylin and eosin (H&E) staining of organs collected at the end of the experiment revealed no major organ-related toxicities (Figure 8f). These data suggested that GA exhibits potent antitumor activity with low toxicity *in vivo*.

## Discussion

Triterpene saponin GA, the primary ingredient of numerous traditional Chinese medicines for cancer,^35, 36^ is the main bioactive and metabolic component *in vivo*.^37, 38^ Numerous studies have indicated that GA plays an important role in suppressing tumorigenesis by inhibiting tumor growth and metastasis.^8, 12, 39^ However, little is known about the molecular mechanisms underlying the anticancer activity of this compound, especially in sarcoma. In the present study, we demonstrated that proliferation of human sarcoma cells is inhibited by GA *in vitro* and *in vivo* via G0/G1 arrest, autophagy, and apoptosis. In cells subjected to persistent and intense stimuli, autophagy exerts a protective effect to maintain normal survival; in the present work, autophagy was induced by ER stress via the IRE1*α*-JNK/c-jun pathway. Furthermore, autophagy or JNK inhibition increased GA-induced apoptosis rate and cell death.

Apoptosis represents an important strategy for the eradication of tumor cells and treatment of cancer. Here, we reveal that GA induces apoptosis through both extrinsic and intrinsic pathways. Surprisingly, as cell death was not found to be entirely prevented by the caspase inhibitor, we examined other caspase-independent pathways. Huang *et al.*^40^ found that AIF plays an important role in GA-induced apoptosis in the absence of caspases. Furthermore, Endonuclease G (Endo G), another apoptogenic protein present in the mitochondrial intermembrane space, translocates to the nucleus and directly cleaves nuclear DNA independent of caspase activity.^41^ Accordingly, we examined the expression of AIF or Endo G: no apparent difference in their expression was detected between the mitochondria and cytosol in GA-treated cells (data not shown).

Autophagy, which serves as another caspase-independent cell death pathway, was investigated. TEM revealed the accumulation of AO and MDC-staining acidic vesicles, upregulation of LC3B-II, and formation of autophagosomes, indicating the induction of autophagy following GA treatment. Furthermore, we investigated GA-induced autophagy and the underlying mechanism. We found that treatment with GA induced a considerable increase in JNK and c-Jun phosphorylation. In addition, the expression of JNK was found to be correlated with that of IRE1. However, the data did not support the conclusion that the activation of the JNK pathway occurred before the induction of ER stress.^42^ Our study demonstrated that GA treatment led to remarkable increases in JNK phosphorylation in sarcoma cells that could, in turn, be reversed by treatment with the JNK inhibitor, SP600125, or with siRNA targeting IRE1. The results of current study show that JNK acts downstream of IRE1. Overall, our data suggest that GA induces autophagy in sarcoma cells, partly through stimulation of the ER stress response via activation of the IRE1/JNK pathway.

The effects of autophagy (prodeath or prosurvival) depend on the cell type involved and stimulator used.^43^ For example, in hepatocellular carcinoma, the anticancer effects of linifanib are enhanced by the inhibition of autophagy; however, in rhabdomyosarcoma, autophagy results in a decrease in the antitumor effects of piperlongumine.^44^ The cytotoxic effects of gefitinib against glioma cells mainly involve cell death through autophagy; however, in lung cancer cells, autophagy induced by gefitinib exerts a cytoprotective effect.^45, 46^ Several studies have suggested that autophagy serves as a cell survival mechanism in GA-treated cancers.^47, 48^ Consistent with these findings, the current study indicated that inhibition of JNK/c-jun pathway or GA-induced autophagy significantly increased apoptosis rates and cell death, suggesting GA- induced cytoprotective autophagy in sarcoma cells. However, IRE1*α* knockdown failed to elicit an increase in GA-induced cell apoptosis (Supplementary Figure S2) that may be attributed to the activation of both autophagy and apoptosis by IRE1*α*. However, the mechanism by which IRE1*α* activates GA-induced apoptosis remains to be determined.

ER stress response-mediated apoptosis and cell death are significantly prevented by the activation of autophagy, thus sustaining cell survival as well as homeostasis. One of the main reasons for the limited effects of chemotherapy drugs is the development of drug resistance. Previous studies have demonstrated that the activation of autophagy following ER stress during chemotherapy is related to the development of drug resistance in cancer.^49^ To our knowledge, no previous in-depth findings related to the mechanisms of action of GA in other cancer cell types have been reported. Here, we showed that autophagy blockage greatly improves the cytotoxic effects of GA in sarcoma cells. Furthermore, the negative effects of autophagy on GA-induced apoptosis in sarcoma cancer cells were found to be abolished by autophagy inhibitors. The present findings suggest that the use of such inhibitors serves to enhance the therapeutic effects of GA and improve cancer prognosis.

In conclusion, the present study is the first to demonstrate that GA effectively inhibits the proliferation of sarcoma cells by causing G0/G1-phase arrest, and leads to cell death by inducing apoptosis. In the osteosarcoma xenograft model, GA was found to elicit significant antitumor activity with low levels of toxicity. Furthermore, in sarcoma cells, the IRE1 pathway appears to play a critical role in ER stress-activated autophagy following GA treatment; this counters ER stress-induced apoptosis and exerts protective effects against cell death. The blockage of autophagy or the JNK pathway enhances the antiproliferative effect of GA. Our results suggest that targeting the autophagy pathway in combination with GA treatment serves as a novel strategy for sarcoma therapy.

## Materials and methods

### Cell culture

HOS human osteosarcoma cells (ATCC: CRL-1543) and HT1080 human fibrosarcoma cells (ATCC: CCL-121) were obtained from the American Type Culture Collection (ATCC, Manassas, VA, USA). The cells were cultured in Eagle’s minimum essential medium (MEM) (Gibco BRL, Grand Island, NY, USA) supplemented with 10% (v/v) fetal bovine serum (FBS) and 1% (v/v) antibiotics (100 U/ml penicillin and 100 *μ*g/ml streptomycin). The cells were maintained in an incubator set to 37 °C with 5% CO_2_.

### Reagents and antibodies

GA was acquired from the National Institutes for Food and Drug Control in Shenzhen, Guangdong, China. The 3-(4,5-dimethylthiazol-2-yl)-2,5-diphenyltetrazolium bromide (MTT), SP600125, chloroquine (CQ), and DMSO were obtained from Sigma (St. Louis, MO, USA). MEM medium, FBS, penicillin, streptomycin, and phosphate-buffered saline (PBS) were purchased from Gibco Life Technologies (Grand Island, NY, USA). Hoechst 33342 was obtained from Molecular Probes (Grand Island, NY, USA). Primary antibodies, including cleaved-poly (ADP-ribose) polymerase (PARP), Beclin1, p62, LC3, p-JNK (Thr183/ Tyr185), JNK, p-c-jun (ser63), c-jun, IRE1*α*, together with GAPDH antibodies and secondary antibodies, were purchased from Cell Signaling Technology, Inc. (Beverly, MA, USA). 3MA were purchased from Selleckchem (Houston, TX, USA). Antibodies against caspase-3, -8, and -9, Bcl-2, Bcl-xl, survivin, Cyclin D1, Cyclin E, and Cdk4 were purchased from Abcam (Cambridge, England).

### MTT assay

MTT assay was employed to examine the effects of GA on the proliferation of sarcoma cells. Briefly, the cells were seeded in 96-well plates at 2 × 10^3^ cells/well in 200 *μ*l medium. Then, the cells in the wells were treated with various concentrations of GA and cultured for 24, 48, or 72 h. At the end of culture, MTT solution (0.5 mg/ml in 20 *μ*l PBS) was added to each well and incubated for 4 h at 37 °C. An Erlotinib mesylate enzyme-labeled instrument (Thermo, Waltham, MA, USA) was used to measure the absorbance of each well at 570 nm. Data were calculated from three independent experiments, each performed in sextuplicate.

### Detection of acidic vesicular organelles

Formation of acidic vesicular organelles (AVOs), a morphological characteristic of autophagy, was detected by acridine orange (AO) staining. Cells were stained with 1 *μ*g/ml acridine orange for 20 min and the samples were observed under a laser scanning confocal microscopy (excitation, 546 nm; emission, 575/640 nm).

### Visualization of autophagic vacuoles

The autofluorescent agent MDC was used as a specific autophagolysosome marker to analyze the autophagic process. Sarcoma cells were treated with different concentrations of GA for 24 h. Autophagic vacuoles were labeled with MDC by incubating cells with 50 *μ*M MDC in PBS at 37 °C for 20 min. After incubation, cells were washed three times with PBS and immediately analyzed by a laser scanning confocal microscopy (excitation, 390 nm; emission, 460 nm).

### Colony-formation assay

Cells were seeded in six-well plates at a density of 1000 cells per well. In the drug treatment group, the medium was changed with fresh medium containing GA (10–40 *μ*M) for ∼14 days until the cells grew to visible colonies. Colonies were fixed with 4% paraformaldehyde and stained by crystal violet for 15 min at room temperature. The colonies that consisted of 450 cells were counted.

### Transwell migration assay

Transwell assay was conducted as described previously with some modifications. Serum-free medium was added to the top chamber of 24-well transwell plate (Millipore, Plano, TX, USA), and the bottom chambers were filled with 600 *μ*l DMEM medium containing various growth factors. The top chambers were seeded with 100 *μ*l DMEM medium and sarcoma cells (4 × 10^4^ cells per well). Immediately, 100 *μ*l DMEM medium with various concentrations of GA was added to the upper chamber. After 24 h, migrated cells were fixed in 4% paraformaldehyde and stained with 0.05% crystal violet. Images were taken using a ZEISS (Heidenheim, Germany) digital microscope and invading cells were counted by manual counting. The assays were replicated three times.

### Cell cycle analysis by flow cytometry

Cells were seeded in six-well plates with a density of 1 × 10^6^/ml and then treated with GA at different concentrations for 24 h. After GA treatment, the cells were harvested, washed with PBS, and fixed with cold 75% ethyl alcohol at 4 °C overnight. The cells were then again washed with PBS and incubated with RNase A for 30 min followed by staining with 500 *μ*l propidium iodide for 30 min at room temperature. Cell cycle analysis was performed on the Accuri C6 (BD Biosciences, Mountain View, CA, USA).

### Morphological apoptosis

Cells were cultured at a density of 1 × 10^5^/ml per well on coverslips in 6-well plates and then treated with 10–40 *μ*M GA for 24 h. After incubation, cells were washed twice with PBS, fixed with 4% paraformaldehyde for 30 min, and then stained with Hoechst 33342 solution (5 *μ*g/ml) for 10 min in the dark at 37 °C. Cells were assessed by fluorescence microscope for morphological changes of the nucleus.

### Mitochondrial membrane potential assay

The JC-1 Assay Kit (Beyotime, Beijing, China) was used to measure the alteration of mitochondrial membrane potential according to the manufacturer’s instructions. Cells were seeded in 6-well plates with a density of 5 × 10^5^/ml and then treated with GA at concentrations ranging from 10 to 40 *μ*M for 24 h. Then, 100 *μ*l of JC-1 staining solution was added into 1 ml of culture medium and incubated for 20 min at 37 °C in a CO_2_ incubator. The samples were analyzed by flow cytometry, and JC-1 aggregate was measured at the FL-2 channel and green fluorescent (both JC-1 monome) at the FL-1 channel (BD Biosciences).

### Apoptosis analysis by flow cytometry

Cells were seeded in 6-well plates with a density of 1 × 10^6^/ml and then treated with GA at concentrations ranging from 0 to 40 *μ*M for 24 h. After GA treatment, cells were harvested, washed twice with cold PBS, and resuspended in the 1 × binding buffer. Then, cells were incubated with FITC-conjugated annexin V and PI for 15 min in the dark at room temperature, and the samples were analyzed using the flow cytometry in 1 h (BD Biosciences).

### Western blotting analysis

Cells were cultured in 6-well plates at a density of 5 × 10^5^/ml per well and then treated with GA (0–40 *μ*M) for 24 h. Cells were washed with PBS and lysed in ice-cold RIPA containing a protease and a phosphatase inhibitor cocktail for 30 min on ice. Cell lysates were centrifuged at 13 000 × *g* for 15 min at 4 °C, and the supernatant was collected. Protein concentrations were quantified using the BSA Protein Assay (Thermo) according to the manufacturer’s instructions. Equal amounts (30 *μ*g) of total protein were separated by SDS-PAGE (8–12%) at 100 V for 1.5 h and transferred to 0.45 *μ*m PVDF membrane at 100 V for 1 h. After blocking with 5% nonfat milk in PBST buffer for 1 h at room temperature, the membranes were incubated with primary antibody at 4 °C overnight. The membranes were washed three times with PBST buffer and then incubated with peroxidase-conjugated secondary antibody for 1 h at room temperature. Specific antibody binding was detected by the Chemiluminescence Kit (Millipore).

### Orthotopic xenograft

#### OS mouse model

Female BALB/c-nu mice (Shanghai Slac Laboratory Animal Co., Ltd, Shanghai, China) were purchased at 4 weeks of age and housed in a standard animal laboratory with free access to water and food. HOS cells were digested and washed by cold PBS for three times, and the final concentration was 1 × 10^7^/ml in cold PBS. A volume of 100 *μ*l cell suspension was injected into medullary cavity of tibia. When the tumors in the tibia were macroscopic, mice were randomly divided into three groups: control group and two GA group (six mice in each group). Control group received intraperitoneal injection of 100 *μ*l 5% DMSO every other day, whereas GA group was injected with 100 *μ*l GA (20 or 40 mg/kg, diluted with 5% DMSO). After seven times of drug administration, the mice were killed, and the tumors were removed, weighted, and fixed for use in immunohistochemical experiments. All the animal-related procedures were approved by the animal care and use committee of Sir Run Run Shaw Hospital.

### TUNEL assay

Apoptosis detection was identified using a TUNEL Assay Kit (Beyotime) according to the manufacturer’s instructions. In brief, paraffin-embedded slides were deparaffinized with xylene and ethanol and rehydrated cell by proteinase K. After several washes with PBS, sections were incubated with TUNEL reaction mixture freshly prepared for 1 h at 37 °C in a moist chamber. Apoptotic cells on the slides were observed under an Olympus light microscope (Olympus, Tokyo, Japan) in randomly chosen fields.

### Histopathology and immunohistochemistry

Formalin-fixed tissue samples were embedded in paraffin and 4 *μ*m sections were cut. Primary tumors, heart, liver, spleen, lung, and kidney sections were stained with H&E for routine histological examinations and morphometric analysis. For immunohistochemical staining, slides were deparaffinized in xylene and rehydrated with graded alcohol and incubated in 3% hydrogen peroxide to block the endogenous peroxidase activity. Antigen retrieval was performed by boiling the slides in 10 mM sodium citrate (pH 6.0) for 30 min. Then, slides were blocked in 10% normal goat serum for 15 min, followed by incubation with p-JNK, and cleaved caspase-3 at 4 °C overnight in a moist chamber. On the next day, slides were washed in PBS and incubated with the second antibody for 1 h at room temperature. Immunoreactivity was detected using the Vectastain Elite DAB KIT (Vector Laboratories, Burlingame, CA, USA).

### Statistical analysis

Statistical analysis was performed using the SPSS version 18.0 software (IBM Corporation, Chicago, IL, USA). Student’s *t*-test, Fisher’s exact test, and one-way ANOVA were used for calculating the significance between different groups. Statistical significance is indicated by *P*<0.05. All data were expressed as mean±S.D. of three independent experiments.
