# Supplementary figures and images for: Blocking autophagy enhances the apoptotic effect of 18β-glycyrrhetinic acid on human sarcoma cells via endoplasmic reticulum stress and JNK activation
Source: Cell Death Dis. 2017 Sep 21;8(9):e3055–. doi: 10.1038/cddis.2017.441 (PMC5636985; doi:10.1038/cddis.2017.441)

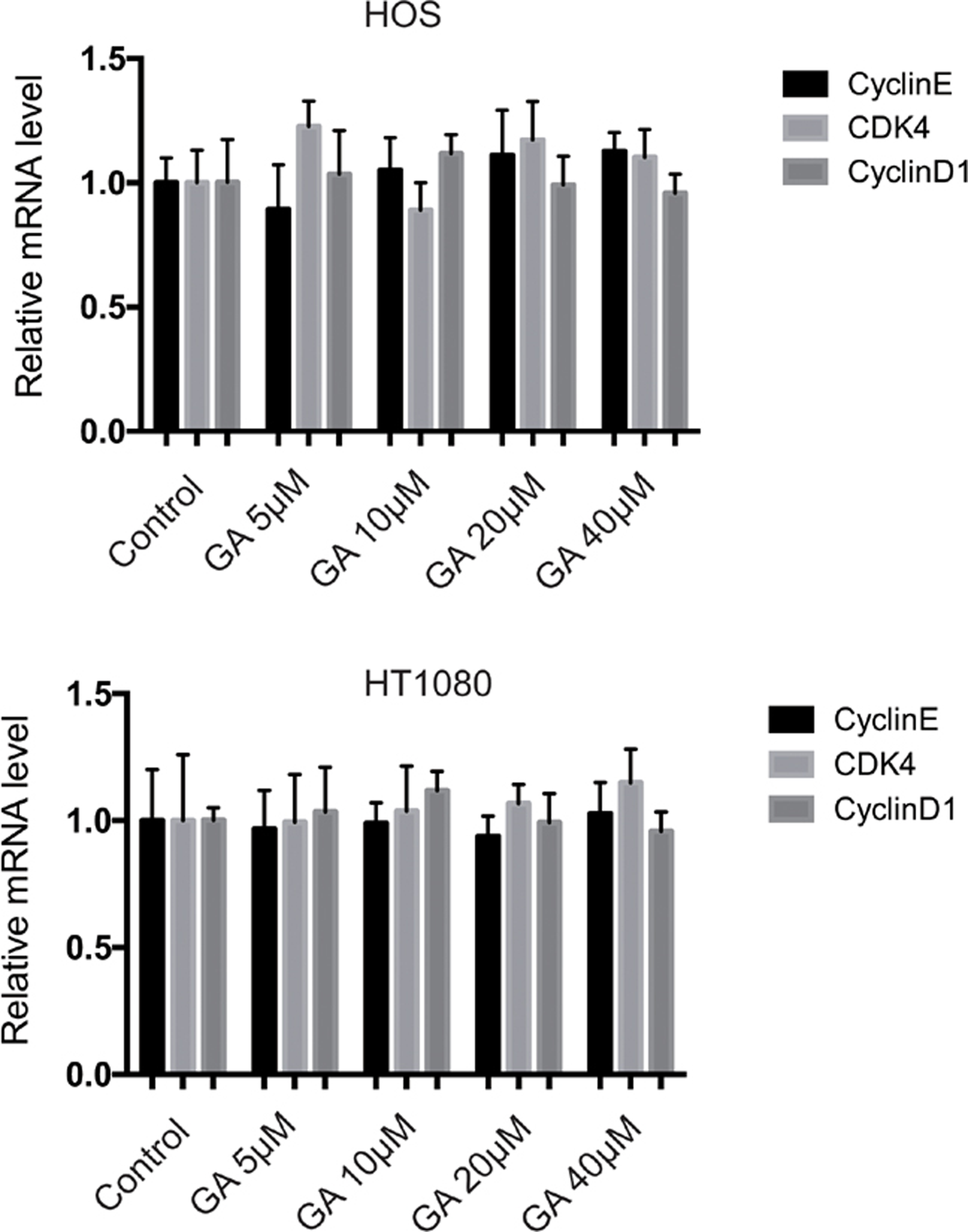

Supplement: Supplementary Figure S1 [file cddis2017441x1.tif]

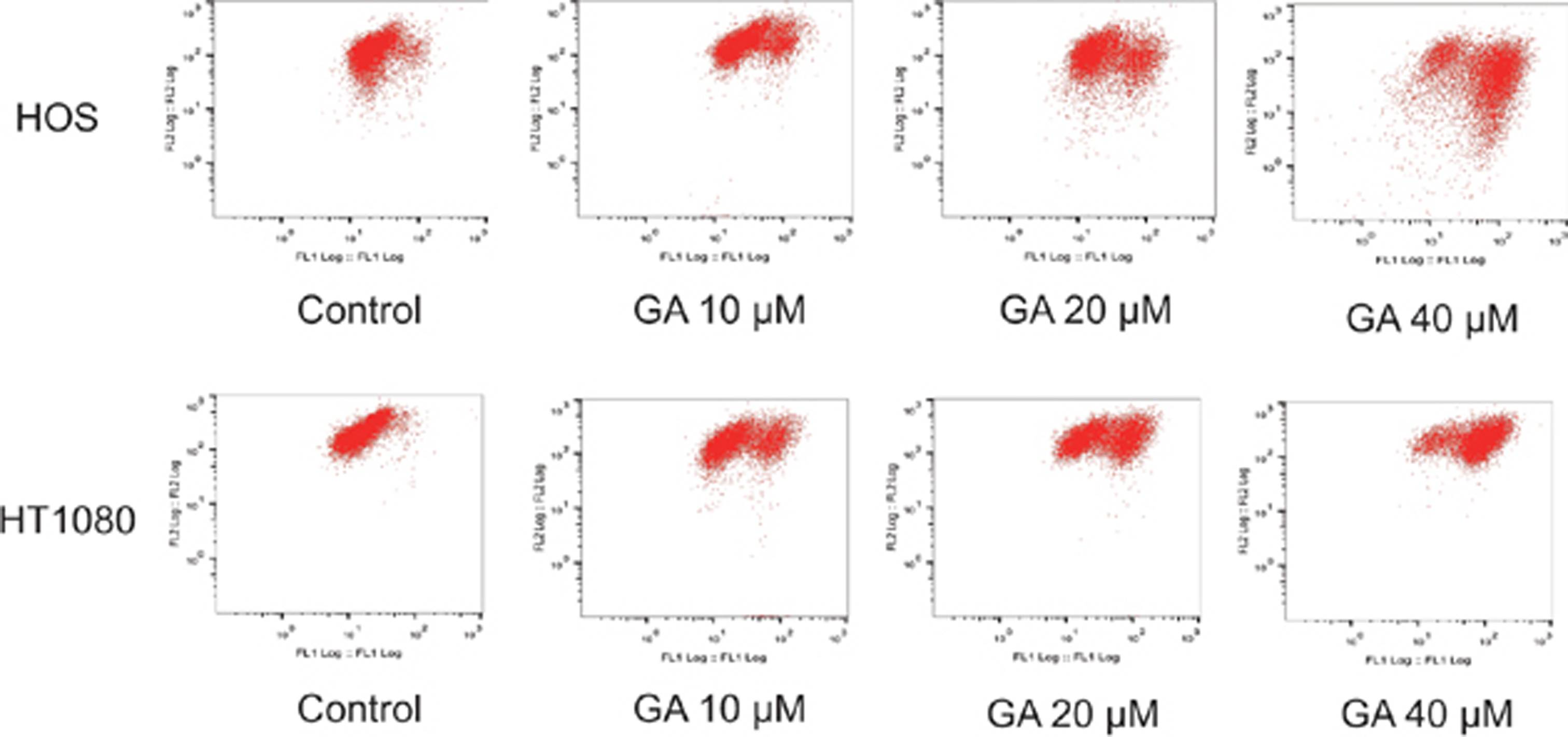

Supplement: Supplementary Figure S2 [file cddis2017441x2.tif]

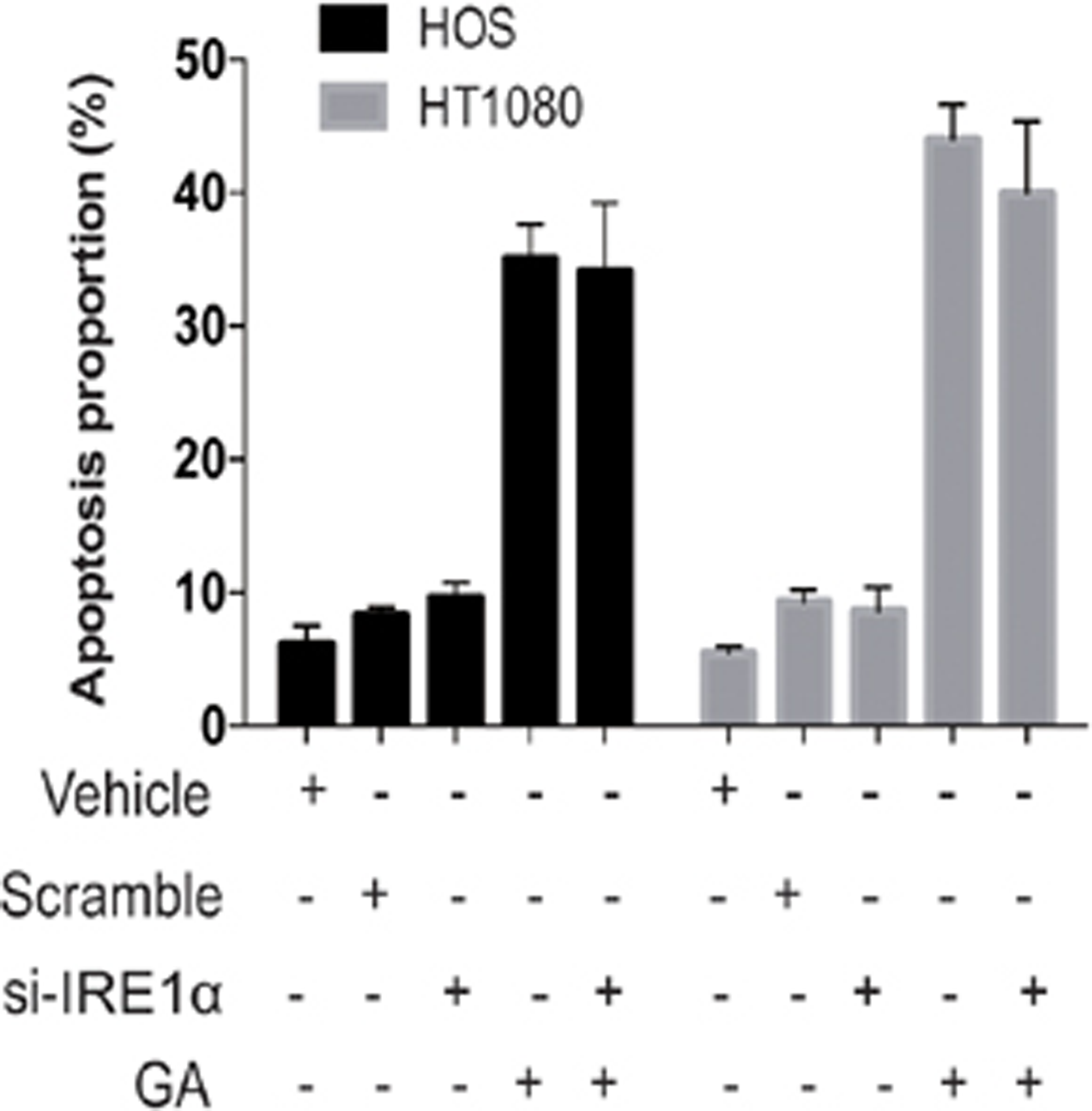

Supplement: Supplementary Figure S3 [file cddis2017441x3.tif]

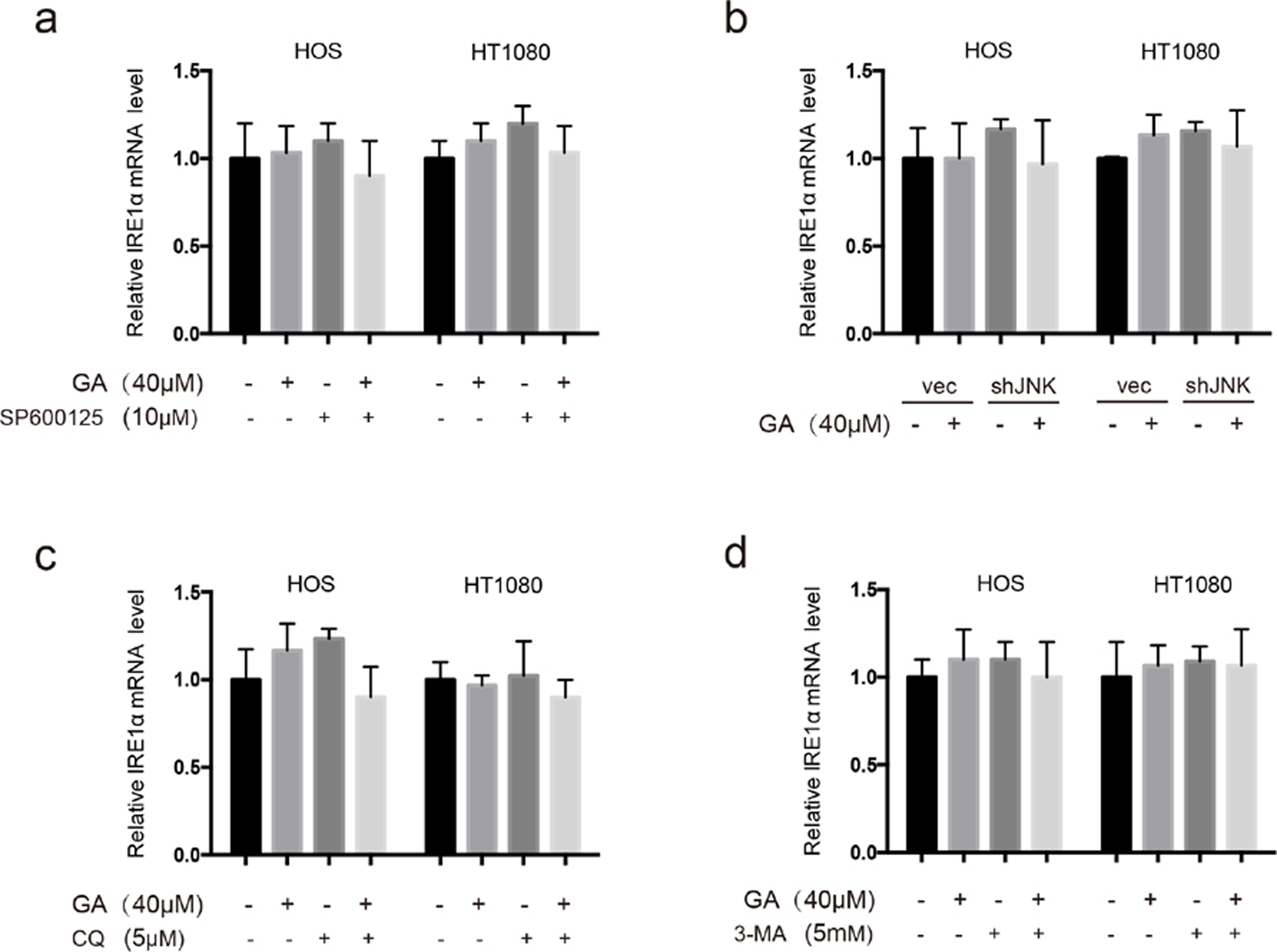

Supplement: Supplementary Figure S4 [file cddis2017441x4.tif]
