# Supplementary material for: Blocking autophagy enhances the apoptotic effect of 18β-glycyrrhetinic acid on human sarcoma cells via endoplasmic reticulum stress and JNK activation
Source: Cell Death Dis. 2017 Sep 21;8(9):e3055–. doi: 10.1038/cddis.2017.441 (PMC5636985; doi:10.1038/cddis.2017.441)
Supplement: Supplementary Figure Legends [file cddis2017441x5.docx]

**Figure S1. The mRNA levels of cell cycle markers by GA treatment.** Cells were treated with control or GA for 24 h and the mRNA levels of cell cycle markers were analyzed by qRT-PCR.

**Figure S2. The induction of mitochondrial depolarization by GA in sarcoma cells.** The mitochondrial membrane potential was measured with JC-1 fluorescent probe and assessed by flow cytometry.

**Figure S3. Effect of IRE1α knockdown on GA-induced cell apoptosis.** After knock down of IRE1α by siRNA, HOS and HT1080 cells were incubated with or without 40 μM of GA for 24 h. Cells were analyzed by flow cytometry. Histograms were shown for analyzed cells (n=3).

**Figure S4. Effect of JNK/c-jun and autophagy inhibition on IRE1α mRNA level in sarcoma cells.** (a) After suppression of the JNK/c-jun cascade by SP600125 (10 μM, 1 h) pretreatment, HT1080 and HOS cells were treated with GA (40 μM) for 24 h. MRNA expression levels were detected by qRT-PCR. (b) After suppression of the JNK by shRNA transfection, HT1080 and HOS cells were treated with GA (40 μM) for 24 h. MRNA expression levels were detected by qRT-PCR. (c) HT1080 and HOS cells were treated with GA (40 μM) for 24 h with or without pretreatment of CQ (5 μM, 1 h). MRNA expression levels were detected by qRT-PCR. (d) HT1080 and HOS cells were treated with GA (40 μM) for 24 h with or without pretreatment of 3-MA (5 mM, 2 h). MRNA expression levels were detected by qRT-PCR.
